# High-Order Polynomial Fitting Assistance for Fast Double-Peak Finding in Brillouin-Distributed Sensing

**DOI:** 10.3390/s21010187

**Published:** 2020-12-30

**Authors:** Marcelo A. Soto, Alin Jderu, Dorel Dorobantu, Marius Enachescu, Dominik Ziegler

**Affiliations:** 1 Department of Electronic Engineering, Universidad Técnica Federico Santa María, 2390123 Valparaíso, Chile; marcelo.sotoh@usm.cl; 2 NanoPRO START MC S.R.L., 041337 Bucharest, Romania; alin.jderu@cssnt-upb.ro (A.J.); dorel.dorobantu@cssnt-upb.ro (D.D.); dominik.foslab@gmail.com (D.Z.); 3 Center for Surface Science and Nanotechnology (CSSNT), University Politehnica Bucharest, 060042 Bucharest, Romania

**Keywords:** fiber optics sensors, distributed Brillouin sensors, stimulated Brillouin scattering

## Abstract

A high-order polynomial fitting method is proposed to accelerate the computation of double-Gaussian fitting in the retrieval of the Brillouin frequency shifts (BFS) in optical fibers showing two local Brillouin peaks. The method is experimentally validated in a distributed Brillouin sensor under different signal-to noise ratios and realistic spectral scenarios. Results verify that a sixth-order polynomial fitting can provide a reliable initial estimation of the dual local BFS values, which can be subsequently used as initial parameters of a nonlinear double-Gaussian fitting. The method demonstrates a 4.9-fold reduction in the number of iterations required by double-Gaussian fitting and a 3.4-fold improvement in processing time.

## 1. Introduction

For about three decades, Brillouin scattering has been widely used in the development of distributed optical fiber sensors. Exploiting the Brillouin acousto-optic interaction between optical signals and the optical fiber material, a distributed temperature or strain profile along an optical fiber can be obtained for sensing purposes [1,2]. The environmental information is contained on the peak frequency of the Brillouin gain spectrum (BGS), so-called Brillouin frequency shift (BFS) of the sensing fiber. In the time-domain approach, the BGS is measured using optical pulses, whose length determines the spatial resolution of the sensor [2]. To extract the local temperature or strain information over the sensing optical fiber, measurements are post-processed to find the frequency of the BGS maximum amplitude at each fiber location [3,4,5,6,7].

Although several signal processing approaches have been proposed to estimate the BFS [3,4,5,6,7], curve fitting techniques [6,7] (e.g., using a Gaussian, Lorentzian, Voigt or quadratic curve) are still the most common procedure to retrieve the local BFS in a distributed sensor. Single-peak curve fitting techniques provide a reliable and precise extraction of the BFS in many scenarios. However, they fail to return a reliable BFS estimation when the measured Brillouin spectrum exhibits more than one resonance peak. Measurements with multiple Brillouin spectral peaks can stem from several practical reasons: (i) the use of an optical fiber with more than one acoustic mode [8], (ii) the use of a pump pulse exhibiting a poor extinction ratio [9], or (iii) the occurrence of sharp strain or temperature longitudinal transitions [10,11,12]. In the first case, when the sensing fiber has two Brillouin spectral peaks, single-peak fitting methods can easily result in unreliable BFS extraction, eventually requiring more sophisticated curve fitting algorithms to increase the reliability in the BFS estimation. In the second case, optical pulses with poor extinction ratio result in residual continuous-wave light that is launched into the sensing fiber [9], leading to measurements with a Brillouin gain peak that does not contain any spatial information (i.e., containing the integrated mean information of the entire fiber), which typically overlaps and occludes the useful peak containing local information. Finally, when sharp longitudinal BFS changes occur within a fiber section equal or shorter than the spatial resolution, two spectral peaks will locally appear even if the sensing fiber has only a single BGS resonance [10,11,12]. To increase the reliability in the BFS retrieval, double-peak fitting [11,12] could be applied to all mentioned circumstances. This can be performed by adjusting, for instance, the parameters of two Lorentzian, pseudo-Voigt or Gaussian curves to fit the measured spectral data points using a nonlinear least-square (NLS) regression. However, such fitting approach requires an iterative procedure that may involve prohibitively long processing times, especially when fast measurements are required along with a large number of sampled points over the entire sensing fiber.

In this paper, we propose and validate the use of a high-order polynomial fitting to obtain an initial estimation of the frequencies of the two Brillouin peaks, with the aim of reducing the computation time of nonlinear double-Gaussian fitting methods. Compared to the conventional approach, which in the best situation uses the intrinsic BFS of the non-perturbed fiber as an initial parameter, our method reduces the numbers of iterations and the computation time of double-Gaussian fitting based on NLS regression using the Levenberg–Marquardt algorithm [13]. Experimental results point out that, when the two Brillouin peaks do not spectrally overlap, the method proposed here has also the potential to directly provide the two local Brillouin frequency values without the need of further double-Gaussian curve fitting.

## 2. Materials and Methods

**Figure 1:**
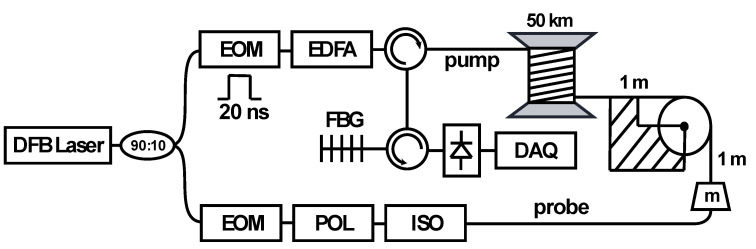
Experimental setup of a standard Brillouin optical time-domain analysis (BOTDA) sensor over a 50 km-long sensing fiber. A short and sharp strain variation is applied along a 2-m-long section near the far fiber end.

To test the proposed fitting procedure, a standard layout of a Brillouin optical time-domain analysis (BOTDA) system is used, as shown in Figure 1. This setup employs a 10 mW distributed feedback (DFB) laser operating at 1550 nm, whose light is split into two branches. In the upper branch (10%), an electro-optic modulator (EOM) with high extinction ratio is used to generate 20 ns optical pulses (providing 2 m spatial resolution) with about 40 dB extinction ratio, which is required to eliminate any continuous-wave Brillouin response along the sensing fiber [2]. Optical pulses are then boosted by an erbium-doped fiber amplifier (EDFA) and launched into the sensing fiber through an optical circulator with an optical power of ~100 mW (limit imposed by the onset of nonlinear effects such as modulation instability [14]). The lower branch (90%) is used to generate a two-sideband suppressed-carrier probe signal by using another EOM, driven by a microwave signal. A polarization switch (POL) is then inserted in the generation of the probe signal, allowing us to consecutively send a probe signal with two orthogonal polarizations, so that the presence of polarization fading in the measured Brillouin traces could be eliminated. The probe signal, after interacting with the pump pulse in the sensing fiber through stimulated Brillouin scattering, is launched into a detection stage conformed by a circulator and a narrowband fiber Bragg grating (FBG) filter. This FBG filter is used to select the lower-frequency probe sideband and filter out unwanted spectral components like the upper-frequency probe sideband and the Rayleigh backscattered light generated by the pump pulse along the sensing fiber. Then, a 300 MHz photodetector and a fast data acquisition (DAQ) system are used to measure the Brillouin time-domain traces.

**Figure 2:**
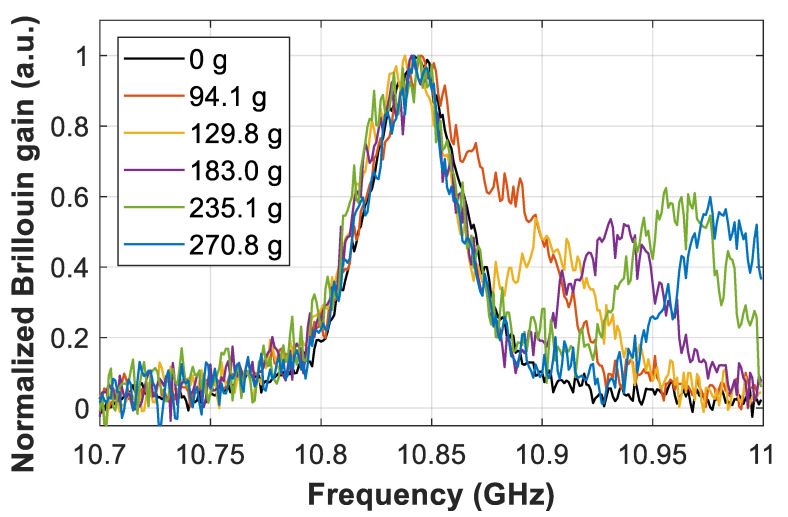
Brillouin spectral measurements obtained at the transition fiber section where strain is applied. Several strains are applied to induce different spectral separations among local Brillouin peak resonances. For the sake of clarity, spectral measurements shown here are obtained with 13 dB SNR.

To evaluate the proposed high-order polynomial fitting method, we induce a local double Brillouin spectral peak by applying a sharp longitudinal strain variation at the end of a 50-km-long sensing fiber. As shown in Figure 1, this is achieved by applying different weights to a 2-m-long section near the far end of the fiber. To analyze the different realistic spectral conditions (i.e., different separations between the two local BGS peaks), several distinct masses have been used, which are: 0 g, 94.1 g, 129.8 g, 183.0 g, 235.1 g, and 270.8 g. Knowing the typical Young’s modulus of silica standard optical fibers, which leads to a mass-to-strain conversion factor of 11.0 με/g [15], the applied masses correspond to applied strains of 0 mε, 1.04 mε 1.43 mε, 2.01 mε, 2.59 mε, and 2.98 mε. Note that the length of the stretched fiber is equal to the spatial resolution of the sensor, and therefore longitudinal transitions of the measured BGS clearly exhibit two resonance peaks, as depicted in Figure 2. In a very ideal scenario, only the midpoint of the strained fiber section would exhibit a single BGS, which might not be always the case in real implementations using the optical pulses with limited extinction ratio. For the sake of clarity, Figure 2 shows Brillouin spectral measurements obtained within the BGS transition with a signal-to-noise ratio (SNR) of 13 dB at 50 km distance, obtained with 8 k averaged traces and 1 MHz scanning step.

**Figure 3:**
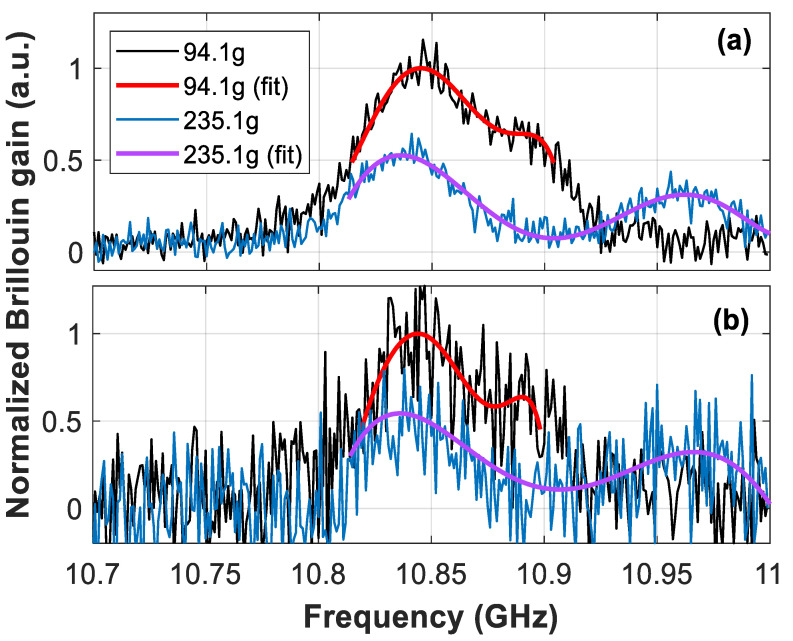
Sixth-order polynomial fitting of two Brillouin spectral measurements at the transition fiber section, obtained by small (94.1 g) and large (235 g) applied strains (masses), for (**a**) 10 dB SNR and (**b**) 5 dB SNR.

Note that BGS measurements exhibit different spectral behavior depending on the applied strain. Thus, for small weights (e.g., mass of 94.1 g) two overlapping BGS can be observed; while for larger weights (e.g., 235.1 g and 270.8 g), the two spectral peaks are clearly separated. Polynomial curves of different orders have been fitted to each of the measured spectra. While the conventional second-order curve only fits one resonance peak [6], polynomial curves with order 5 or higher show to provide a reliable matching with the measured spectral shape. Figure 3a shows the BGS measured at the transition fiber section for the case of applying 94.1 g and 235 g, when having measurements with 10 dB SNR, along with the respective fitted sixth-order polynomial curves. It must be mentioned that the use of higher order polynomials might result in overfitting of the measured shape, eventually leading to a fitted curve shape that may be highly influenced by the noise realization in each measurement. Note that low-order polynomial curves cannot always secure a correct spectral fitting because of the low degree of freedom (i.e., the existence of only a few coefficients), especially when the two BGS peaks are clearly separated. On the other hand, higher-order curves can in principle provide a much better fitting of the dual-peak BGS. This is indeed an interesting feature of the method, giving the flexibility of using high-order polynomial fitting with different Brillouin spectral linewidths and even in the case of spectral asymmetries. However, the presence of noise in the measurements makes fitting curves with higher orders (higher than 6 or 7) much more susceptible to noise, resulting in a fitting curve that may differ from the real BGS shape. Empirical findings during our work have demonstrated that a sixth-order curve behaves well for all the studied cases, even under lower SNR scenarios, as shown in Figure 3b for the case of 5 dB SNR, thus providing a good trade-off between reliable BGS representation and noise filtering (due to the low-pass filtering effect of the fitting process). Note that the SNR has been adjusted to 10 dB and 5 dB by simply changing the number of averaged traces in the experiment, using 2 k averaged traces to get 10 dB SNR at 50 km distance and 200 traces to get 5 dB at the same distance.

**Figure 4:**
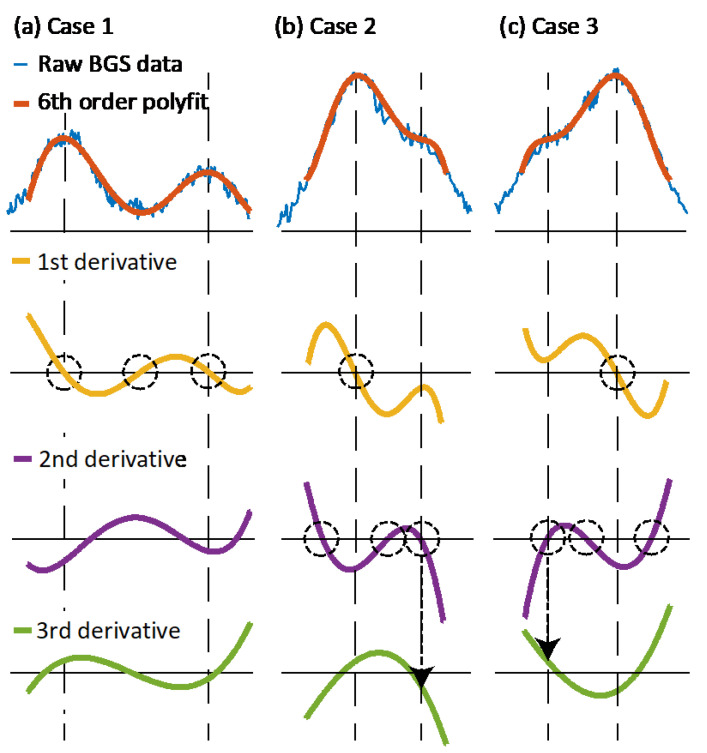
Proposed method for finding a first guess of the Brillouin resonance peaks based on a high-order polynomial curve fitting and its three first order derivatives. There cases are shown: a case with large spectral separation (Case 1), and two cases with overlapping peaks (Cases 2 and 3).

To extract an estimation of each peak frequency in local BGS measurements, the first three derivatives of the fitted curve must be calculated. This procedure is shown in Figure 4, where the derivatives indicate the presence of local maxima or inflection points that give a first estimation of the peak frequencies. Note that the two first-order derivatives are sufficient to detect local maxima when the BGS contains well separated peaks (Case 1 in Figure 4). In this case, the derivative is zero at the peak frequencies, with a negative second derivative. However, when the two peaks overlap (Cases 2 and 3 in Figure 4), the third-order derivative must be used to unambiguously estimate the correct peak frequencies. In this case, besides the frequency at which the first derivative is zero and the second derivative is negative (corresponding to the main peak frequency), secondary peaks are found at spectral positions where the second derivative is zero and the sign of the third derivative coincides with the sign of the first derivative. It must be mentioned that, in Cases 2 and 3, the estimated peak frequencies do not correspond to the exact BFS values, but they represent a good initial guess to be used in a precise nonlinear two-peak Gaussian fitting algorithm.

**Figure 5:**
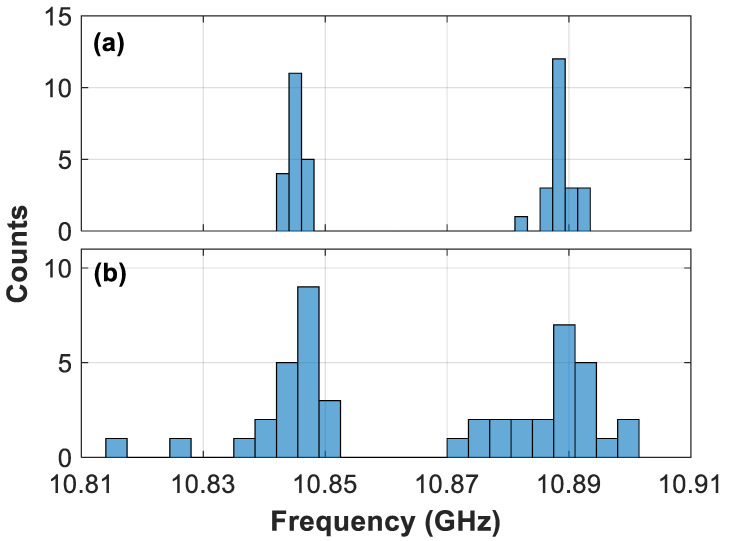
Histogram of the first estimation of the local BFS obtained by the sixth-order polynomial fitting for the case of large applied strain, when having (**a**) 10 dB SNR and (**b**) 5 dB SNR. For the sake of clarity only the case of well-separated peaks is here depicted (i.e., case of applying 235 g) to better illustrate the procedure of the proposed approach. The bin sizes are selected as twice the expected BFS standard deviation for the respective SNR conditions.

It is worth noting that the large degree of freedom given by the seven coefficients of the sixth-order polynomial curve and the presence of noise lead to uncertainties in the initial estimation of the BFS values. These uncertainties are essentially due to the influence of noise on the spectral overfitting that may result from the high-order polynomial curve. To reduce uncertainty in the initial estimated BFS values, it is possible to use the histogram of the peaks found at the local position, which could be calculated within a window moving along the fiber. The histogram allows us to find the most-commonly repeated BFS values and take them as more precise BFS estimations compared to the values directly obtained from fitting. To increase the number of measured data points and obtain reliable histograms, longitudinal oversampling along the traces could be used. If the spatial resolution and noise bandwidth do not lead to a relevant number of independent points, another (or complementary) approach could be the use of consecutive measurements to make the histogram with enough relevant data points. In this case, Figure 5 shows the histogram of the BFS values found within the spatial resolution (using 10 points per spatial resolution, which are found to be independent enough to provide a reliable histogram) and over two adjacent temporal measurements, obtained for both 10 dB and 5 dB SNR. This figure clearly reveals the dominant presence of two peak frequency values, showing that the histogram can help reducing the uncertainty induced by spectral overfitting, and thus improve the precision of the initial BFS estimation. Estimated values are however still uncertain within the bin size of the histograms, which have been here defined as twice the theoretically expected BFS standard deviation [6] for a BOTDA sensor of 2 m spatial resolution (i.e., in this case the bin sizes are 1.3 MHz and 4.2 MHz, for 10 dB and 5 dB SNR, respectively). Note that this BFS standard deviation can be straightforwardly estimated in a real system by knowing some parameters of the sensor, like the SNR and spatial resolution [6].

The estimated BFS values are then used as initial parameters of a double-Gaussian fitting algorithm based on a nonlinear least-square regression using Levenberg–Marquardt algorithm [13]. Similar approach can be followed when fitting a double-Lorentzian or double-Voigt curve, as also commonly used [11,12]. Compared to the conventional approach, in which the initial BFS values of both peaks are assumed as the mean BFS of the fiber, or in the best case as the local BFS obtained during calibration in an unperturbed fiber, the here proposed approach utilizes the BFS values obtained by polynomial fitting as initial parameters to perform NLS regression, reducing the number of computed iterations and convergence time.

## 3. Results

Before evaluating the computation times, we compare the BFS values retrieved by the conventional double-Gaussian fitting (using the unperturbed local BFS of the fiber as initial value) and the ones estimated directly from sixth-order polynomial fitting. Note that the proposed fitting method could be applied to the entire optical fiber, regardless the number of local BGS peaks existing along it. This means that the method does not require a previous knowledge of the BFS distribution, so that the existence of a single or dual BFS values can be obtained automatically by applying process described in Figure 4 and Figure 5 to the entire sensing fiber.

**Figure 6:**
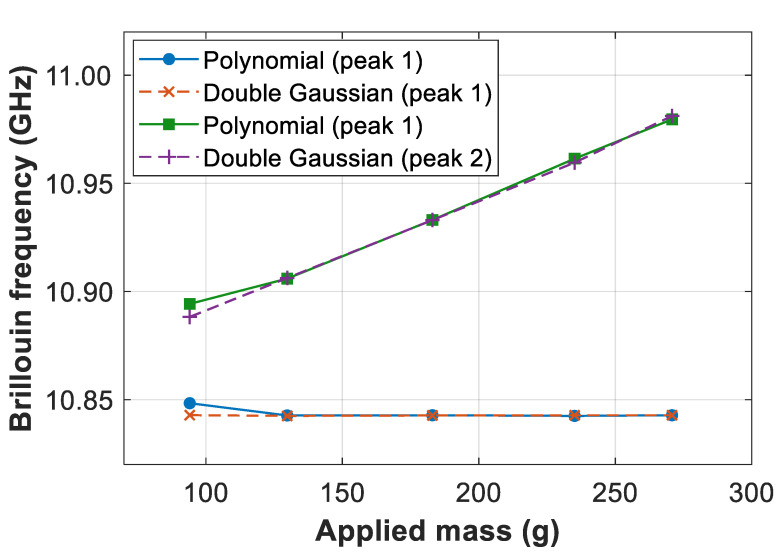
Estimated Brillouin frequencies obtained from 6th-order polynomial fitting and independent double-Gaussian fitting.

Results in Figure 6 point out that the BFS estimations obtained by polynomial fitting match very well the values obtained by nonlinear double-Gaussian fitting, especially under large applied strains (i.e., large masses), when the two BGS peaks do not highly overlap. The obtained BFS values indeed also match the expected Brillouin spectral shifts based on the applied strain and the Brillouin strain coefficient of ~0.05 MHz/με [1,2]. In this large strain range, the evaluated root-mean-square BFS difference is 0.18 MHz and 1.1 MHz for the first and second peaks, respectively. This validates the high precision of the proposed method, implying also that high-order polynomial fitting could be eventually used directly to estimate the frequency of the two local Brillouin peaks without the need of double-Gaussian fitting. Note however that, although the good match verified in Figure 6, a deeper analysis of reliability is still required to assess if high-order polynomial fitting could be used by itself for robust BFS extraction in all practical SNR and spectral conditions. This is however beyond the scope of this paper intended primarily to propose and demonstrate a novel method that reduces the processing time in a double-peak Gaussian fitting approach. Indeed, Figure 6 also shows that when small strains are applied to the fiber and the two local Brillouin peaks highly overlap, e.g., in the case of 94.1 g (see spectrum in Figure 2), the precision of the high-order polynomial fitting turns out to be affected, essentially because the method can only retrieve the envelope of the spectrum. Note that, while the BFS values obtained by double Gaussian fitting represent a precise estimation of the real values (which is partially verified by the linear behavior shown by the dashed lines), the BFS values obtained by polynomial fitting result in differences of 6 MHz and 5.5 MHz for the first (main) and second Brillouin peak, respectively. Those BFS differences could represent high measurand errors if the method is used only to extract the BFS directly, without any other sophisticated processing method that could still be investigated. Nevertheless, the output of the polynomial fitting still represents a better initial frequency to reduce computation time in double-Gaussian fitting algorithms, being the main scope of this work, as it will be verified hereafter.

Then, the impact of the proposed approach is evaluated in terms of the number of iterations and processing time. Fitting is first performed using the local BFS of the unperturbed fiber as initial parameter. The number of iterations and processing time are compared to the ones obtained with the same algorithm but starting from the BFS values estimated by the sixth-order polynomial fitting.

**Figure 7:**
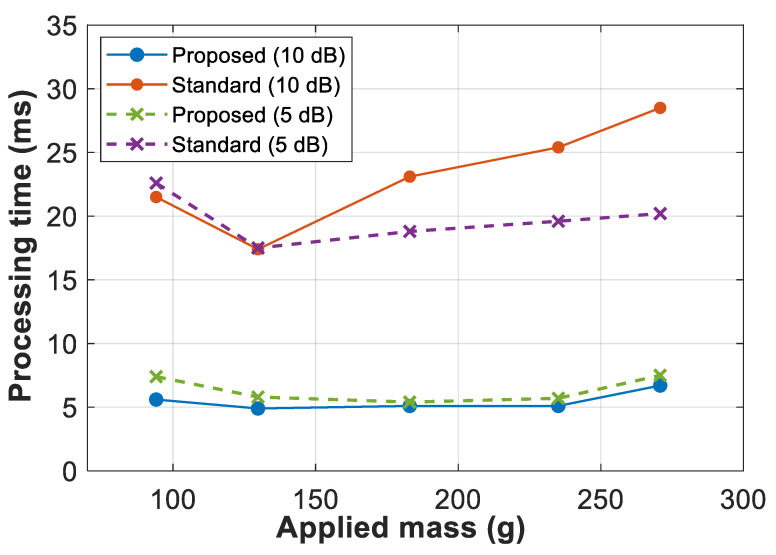
Mean processing time of double-Gaussian fitting based on the standard and proposed approaches, when having 10 dB SNR (straight lines) and 5 dB SNR (dashed lines). Values were obtained averaging the computation time over 100 repeated measurements at each applied mass.

Table 1 shows the average number of iterations and the mean processing time (values in brackets) obtained over 100 consecutive measurements, for each applied strain and SNR. For the sake of visual clarity, a graphical representation of the mean processing time is shown in Figure 7. Results demonstrate that the proposed approach using polynomial-fitting assistance provides an average 4.9-fold reduction in the number of iterations required by double-Gaussian fitting, leading to an average computation time reduction of 3.4 times. Note that the time required for polynomial fitting is negligible compared to the required by the nonlinear NLS regression used by double-Gaussian fitting, thus representing less than the 5% of the computation time used by the conventional double-Gaussian fitting and less than 20% of the time in the proposed approach. This is because high-order polynomial fitting is still considered a linear regression in the coefficients, thus being much faster than the nonlinear iterative NLS approach required by double-Gaussian fitting.

## 4. Discussion

In conclusion, in this paper we have proposed and experimentally validated the use of high-order polynomial fitting to accelerate the processing time of double-Gaussian fitting in distributed Brillouin sensors being affected by existing or artificially induced double Brillouin spectral peaks. Experimental results verify a 4.9-fold reduction in the number of iterations and a 3.4-fold reduction in the processing time of double Gaussian-fitting based on NLS regression using the Levenberg–Marquardt algorithm. A future analysis of reliability and uncertainty of the proposed method is still required to assess the feasibility of using high-order polynomial fitting alone, without the need of Gaussian fitting, to obtain the dual BFS peak values. This must also be compared with an analysis of the BFS uncertainty provided by the double-Gaussian fitting, which, to the best to our knowledge, does not exist today in the literature.

## Figures and Tables

**Table 1:** Comparison of the average number of iterations and computation time (in brackets) of double-Gaussian fitting based on the standard and proposed approaches. Values correspond to the average obtained over 100 measurements for the processing of a single local BGS (at the strained transition location), running Matlab R2019b (Windows 10 Pro) in an Intel(R) Core(TM) i7-9750H CPU @2.6GHz with 32 GB RAM.

SNR	Applied Mass	Standard Approach	Proposed Approach	Enhancement Factor
10 dB	94.1 g	30.6 (21.5 ms)	5.4 (5.6 ms)	5.7 (3.8)
	129.8 g	22.7 (17.4 ms)	4.1 (4.9 ms)	5.5 (3.6)
	183.0 g	23.1 (18.2 ms)	4.1 (5.1 ms)	5.6 (3.6)
	235.1 g	25.4 (20.6 ms)	4.0 (5.1 ms)	6.4 (4.0)
	270.8 g	28.5 (21.6 ms)	6.5 (6.7 ms)	4.4 (3.2)
5 dB	94.1 g	30.9 (22.6 ms)	8.2 (7.4 ms)	3.8 (3.1)
	129.8 g	22.9 (17.5 ms)	5.2 (5.8 ms)	4.4 (3.0)
	183.0 g	23.6 (18.8 ms)	4.6 (5.4 ms)	5.1 (3.5)
	235.1 g	25.1 (19.6 ms)	5.1 (5.7 ms)	4.9 (3.4)
	270.8 g	26.0 (20.2 ms)	7.6 (7.5 ms)	3.4 (2.7)

## Data Availability

The data presented in this study are openly available in FigShare at https://doi.org/10.6084/m9.figshare.13499286, reference number 13499286.
